# Emergence of Hopf bifurcation in an extended SIR dynamic

**DOI:** 10.1371/journal.pone.0276969

**Published:** 2022-10-31

**Authors:** Arash Roostaei, Hadi Barzegar, Fakhteh Ghanbarnejad

**Affiliations:** 1 Department of Physics, Sharif University of Technology, Tehran, Iran; 2 Department of Mathematical Sciences, Sharif University of Technology, Tehran, Iran; 3 Quantitative Life Sciences (QLS), The Abdus Salam International Centre for Theoretical Physics (ICTP), Trieste, Italy; Federal University of Pernambuco: Universidade Federal de Pernambuco, BRAZIL

## Abstract

In this paper, the original SIR model is improved by considering a new compartment, representing the hospitalization of critical cases. A system of differential equations with four blocks is developed to analyze the treatment of severe cases in an Intensive Care Unit (ICU). The outgoing rate of the infected individuals who survive is divided into *nI* and $\frac{bI}{I+b}$ where the second term represents the transition rate of critical cases that are hospitalized in ICU. The findings demonstrate the existence of forward, backward and Hopf bifurcations in various ranges of parameters.

## Introduction

Kermack and Mckendrick [1] developed the first Susceptible-Infectious-Recovered (SIR) model to simulate and predict a disease spread phenomenon and its epidemic state. In this standard model, there are three blocks labeled *S*, *I* and *R* which represent the number or percentage of susceptible, infectious and recovered individuals. The transitions between these compartments are happened by constants *β* and *γ*, respectively indicating the infection rate and the recovery rate.

Since the development of the original SIR model, the parameters *β* and *γ* have been modified in various studies. Some new blocks have been introduced in order to develop better models to more accurately predict the behavior of different epidemics. For instance, *β* was replaced with *kI*^*p*−1^*S*^*q*−1^, *β*^*aS*+*bI*+*cR*^, *μe*^−*mI*^ in [2–4], respectively where the researchers developed the model of individuals’ behavior along with government strategies by considering *β* as a function of other blocks. More recent models on COVID-19 can be found in the articles [5–7].

Moreover, *γ* has been altered in different studies to consider the capacity of the general healthcare system in a country. For instance, *γI* was replaced with:

$$\begin{array}{c} \gamma I=\{\begin{array}{cc} 0\; & I=0\\ rI\; & I>0 \end{array} \end{array}$$ (1)

$$\begin{array}{c} \gamma I=\{\begin{array}{cc} rI\; & 0\leq I\leq I_{0}\\ k\; & I_{0}<I \end{array} \end{array}$$ (2)

$$\begin{array}{c} \gamma I=(\mu_{0}+\left(\mu_{1}-\mu_{0}\right)\frac{b}{b+I})I \end{array}$$ (3)

in the articles [8–10], respectively.

**Fig 1:**
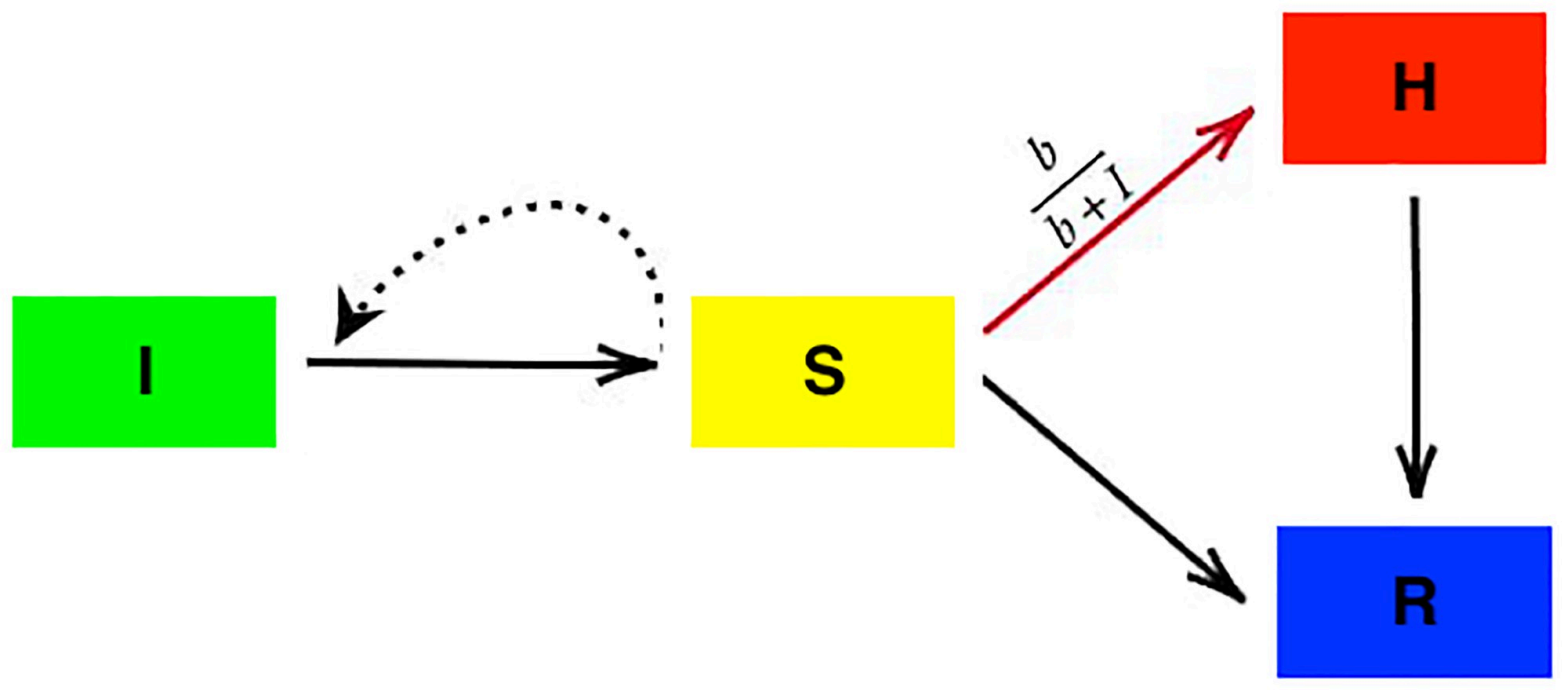
The schematic of the proposed model. It must be emphasized that the natural birth and death are not illustrated here and all the quantities and parameters of the model are listed in Tables 1 and 2.

In our study, an improved SIR model that examines the effects of ICU in hospitals was deployed. The importance of Hospitalization and ICU in real situations can be found also in the articles [11–13]. To this end, a new block *H* was introduced to express the number of individuals in ICU. There is, hence, a new block in the differential equations of the SIR model. Fig 1 represents the schematic of this model with its differential equations expressed as follows:

$$\begin{array}{c} \left\{ \begin{array}{c} \frac{dS}{dt}=A-dS-\beta IS\\ \frac{dI}{dt}=\beta IS-dI-\alpha I-nI-\mu \left(I\right)\\ \frac{dH}{dt}=\mu \left(I\right)-dH-n^{\prime }H\\ \frac{dR}{dt}=nI+n^{\prime }H-dR \end{array}\right. \end{array}$$ (4)

**Table 1:** List of the quantities of the model.

quantities	definition
*S*(*t*)	susceptible individuals
*I*(*t*)	infected individuals
*H*(*t*)	individuals of ICU
*R*(*t*)	recovered individuals

**Table 2:** List of the parameters of the model.

parameter	definition
*β*	infectious rate
*b*	number of beds
*A*	birth rate
*d*	natural death rate
*α*	death rate caused by a disease
*n*	natural recovery rate
*n*′	recovery rate of individuals of ICU

Dimension of the parameters except *b* is *T*^−1^. *b* and the quantities are dimensionless.

In the Eq (4), *A* is the birth rate, *d* is the natural death rate, *α* is the death rate caused by a disease, and *β* is the incidence rate. In this proposed model, individuals of *I* who survive are divided into two groups. The individuals of the first group are cured without hospitalization and do not need special medical equipment utilized in an ICU. This group of patients are transported to the block *R* with the transition rate *nI*. On the other hand, the subjects in the second group need to receive special medical treatments in hospital. These individuals are transferred to a new block *H* with a transition rate *μ*(*I*). The individuals of *H* are recovered with the rate *n*′*H*.

**remark 0.1**. Obviously, the parameters *n* and *n*′ can be functions of *I* and *H* but in order to simplify the equations and future analysis, they are considered as constant. Furthermore, *α*, which is considered as constant like previous argument, represents all the death rate caused by infectious for both of normal and severe patients. One could introduce a different death rate for severe cases which does not actually affect the behavior of the system as we show in future theorems. Therefore, it is more reasonable and convenient to consider just one (average) death rate caused by infectious in block *I* and to concentrate on the transition function to the block *H*, *μ*(*I*), which models the impact of sever patients on behavior of the system and represents the interaction between normal and sever cases.

A function which can model *μ*(*I*) in real situations can be expressed as follows, where *b* represents the number of beds in ICU:

$$\begin{array}{c} \mu_{1}\left(I\right)=\{\begin{array}{c} I\;I\leq b\\ b\;b\leq I \end{array} \end{array}$$

At first, when the number of infected individuals *I* is less than the number of beds *b*, the transition rate to ICU is *I*. For example, there are 100 beds and 50 infected people, so exactly 50 individuals are transported to ICU. However, when the number of infected individuals *I* exceeds the number of beds *b*, the transition rate to ICU is independent of the number of infected individuals and is identified as *b*. For example, there are 150 infected people and 100 beds, so 100 infected people are transferred to ICU.

However, this procedure has a serious shortcoming, as the function is obviously not differentiable at the point *I* = *b*. Thus the following function was selected which is both similar to the previous function and smooth as well:

$$\begin{array}{c} \mu_{2}\left(I\right)=\frac{bI}{I+b} \end{array}$$

Obviously, in the limits *I* → 0 and *I* → ∞, we have *μ*_2_(*I*)→*μ*_1_(*I*) and $\frac{d\mu_{2}}{dI}\to \frac{d\mu_{1}}{dI}$. Therefore, *μ*(*I*) is defined as follows:

$$\begin{array}{c} \mu \left(I\right)≔\frac{bI}{I+b} \end{array}$$

The differential equations are expressed as follows:

$$\begin{array}{c} \left\{ \begin{array}{c} \frac{dS}{dt}=A-dS-\beta IS\\ \frac{dI}{dt}=\beta IS-dI-\alpha I-nI-\frac{bI}{I+b}\\ \frac{dH}{dt}=\frac{bI}{I+b}-dH-n^{\prime }H\\ \frac{dR}{dt}=nI+n^{\prime }H-dR \end{array}\right. \end{array}$$ (5)

In the system (5), the first two equations are independent of the other equations, so just these equations are considered in order to analyze the system (5) and find its fixed points and bifurcations. As such, the essential equations are:

$$\begin{array}{c} \left\{ \begin{array}{c} \frac{dS}{dt}=A-dS-\beta IS\\ \frac{dI}{dt}=\beta IS-\delta I-\frac{bI}{I+b} \end{array}\right. \end{array}$$ (6)

where *δ* is defined as follows:

$$\begin{array}{c} \delta ≔d+\alpha +n \end{array}$$

**remark 0.2**. This article consists of just mathematical analysis and all of the theorems are proved by mathematical analysis and not by numerical simulations. Furthermore, the aim of this article is to explain and research local behavior of the introduced model and does not explain its applications to the real situations with help of numerical analysis. However, there are several numerical simulations related to some proved theorems in order to clarify their possible applications and to give intuition, and not to justify them.

## Fixed points

If we consider the system (6) as $\dot{X}=f\left(X\right)$, we must solve *f*(X*) = 0 in order to find fixed points, then:

$$\begin{array}{c} \left\{ \begin{array}{c} A-dS-\beta IS=0\\ \beta IS-\delta I-\frac{bI}{I+b}=0 \end{array}\right. \end{array}$$ (7)

By solving the second equation, one can find:

$$\begin{array}{c} \left\{ \begin{array}{c} I=0\\ or\\ S=\frac{1}{\beta }\left(\delta +\frac{b}{I+b}\right) \end{array}\right. \end{array}$$

Next by combining the first case and the first equation, one fixed point can be determined as follows:

$$\begin{array}{c} E_{0}=\left(\frac{A}{d},0\right) \end{array}$$

which is the *disease-free equilibrium(DFE)*.

By combining the second case and the first equation, the following quadratic equation can be formulated:

$$\begin{array}{c} \delta I^{2}+\left(b\left(\delta +1\right)+\frac{\delta d}{\beta }-A\right)I+b\left(\frac{d}{\beta }\left(\delta +1\right)-A\right)=0 \end{array}$$ (8)

As it will be discussed in the following sections, it is advisable to define *R*_0_, *basic reproduction number*, as follows:

$$\begin{array}{c} R_{0}≔\frac{\beta A}{d(\delta +1)} \end{array}$$ (9)

Now with this definition, the Eq (8) can be rewritten as follows:

$$\begin{array}{c} P\left(I\right)=c_{2}I^{2}+c_{1}I+c_{0}=0 \end{array}$$ (10)

with the following coefficients:

$$\begin{array}{c} \left\{ \begin{array}{c} c_{2}=\delta \\ c_{1}=b\left(\delta +1\right)+\frac{\delta d}{\beta }-A\\ c_{0}=\frac{bd(\delta +1)}{\beta }\left(1-R_{0}\right) \end{array}\right. \end{array}$$

As a result, the following theorems can be formulated about other fixed points X* ≠ *E*_0_ of the system (6).

**Theorem 0.1**. There is exactly one fixed point X* *if R*_0_ > 1.

*Proof*. It is obvious that:

$$\begin{array}{c} R_{0}>1\Rightarrow \frac{bd(\delta +1)}{\beta }\left(1-R_{0}\right)<0 \end{array}$$

Therefore, the quadratic Eq (10) has two solutions *I*_1_ and *I*_2_ with the condition *I*_1_*I*_2_ < 0. However, the variable *I* must be non-negative in the system (6), so there is just one permissible solution for the quadratic Eq (10) and this proves the theorem.

**Theorem 0.2**. *If* $R_{0}<\frac{\delta }{\left(\delta +1\right)}$, there is no fixed point X*.

*Proof*. It is obvious that *R*_0_ < 1 so *c*_0_ > 0, and we can infer from the assumption that:

$$\begin{array}{cc} R_{0}<\frac{\delta }{\left(\delta +1\right)}\Rightarrow & \frac{A\beta }{d(\delta +1)}<\frac{\delta }{\left(\delta +1\right)}\\ \Rightarrow & \frac{A\beta }{d}<\delta \\ \Rightarrow & 0<\frac{d\delta }{\beta }-A\;\&\;0<b\left(\delta +1\right)\\ \Rightarrow & 0<b\left(\delta +1\right)+\frac{\delta d}{\beta }-A\\ \Rightarrow & 0<c_{1} \end{array}$$

On the one hand, if there are real solutions for the quadratic Eq (10), both of them must be negative (*c*_1_ > 0 & *c*_0_ > 0). On the other hand, there are no real solutions for this equation. Therefore, the theorem has been proved.

**Theorem 0.3**. *If* $\frac{\delta }{\left(\delta +1\right)}<R_{0}<1$, *there are the following cases*:

$b>\frac{d}{\beta }\left(\frac{1}{\delta +1}\right)$ : there is no fixed point X*.

$b<\frac{d}{\beta }\left(\frac{1}{\delta +1}\right)$ : there are two fixed points X* *when R*_0_ *is close enough to 1*.

*Proof*. If *R*_0_ < 1, it is obvious for the two cases that 0 < *c*_0_. Now we can derive the following result for the first case:

$$\begin{array}{cc} & \frac{d}{\beta }\left(\frac{1}{\delta +1}\right)<b\Rightarrow \frac{d}{\beta }<b\left(1+\delta \right)\\ \Rightarrow & \frac{(1+\delta )d}{\beta }<b\left(1+\delta \right)+\delta \frac{d}{\beta }\\ \Rightarrow & \frac{(1+\delta )d}{\beta }-A<b\left(1+\delta \right)+\delta \frac{d}{\beta }-A\\ \Rightarrow & \frac{(1+\delta )d}{\beta }\left(1-\frac{\beta A}{d(\delta +1)}\right)<c_{1}\\ \Rightarrow & \frac{(1+\delta )d}{\beta }\left(1-R_{0}\right)<c_{1}\;\&\;0<1-R_{0}\\ \Rightarrow & 0<c_{1} \end{array}$$

As *c*_1_ > 0 and *c*_0_ > 0, the proof of this case is similar to the previous theorem. Thus there is no fixed point X* in this case.

The second case is somewhat complicated. At first, the new parameter *ϵ* is defined as follows:

$$\begin{array}{c} \epsilon ≔\frac{d(\delta +1)}{\beta }-A \end{array}$$

*ϵ* is positive, because:

$$\begin{array}{cc} R_{0}<1\Rightarrow \frac{\beta A}{d(\delta +1)} & <1\Rightarrow A<\frac{d(\delta +1)}{\beta }\\ 0 & <\epsilon \end{array}$$

and the following properties are obvious:

$$\begin{array}{cc} & A=\frac{d(\delta +1)}{\beta }-\epsilon \\ & \epsilon \to 0^{+}\Leftrightarrow R_{0}\to 1^{-} \end{array}$$

We claim if *ϵ* is small enough, *c*_1_ < 0:

$$\begin{array}{cc} c_{1}<0\Leftrightarrow & b\left(\delta +1\right)+\frac{\delta d}{\beta }-A<0\\ \Leftrightarrow & 0<A-b\left(\delta +1\right)-\frac{\delta d}{\beta }\\ \Leftrightarrow & 0<\frac{d(\delta +1)}{\beta }-\epsilon -b\left(\delta +1\right)-\frac{\delta d}{\beta }\\ \Leftrightarrow & \epsilon <\frac{d}{\beta }-b\left(\delta +1\right)\\ \Leftrightarrow & \epsilon <\left(\delta +1\right)\left(\frac{d}{\beta (\delta +1)}-b\right) \end{array}$$

However, as $b<\frac{d}{\beta (\delta +1)}$ in the second case, the right side of the last inequality is positive, so *ϵ* can be found. As a result:

$$\begin{array}{c} c_{1}<0\Leftrightarrow \epsilon <\left(\delta +1\right)\left(\frac{d}{\beta (\delta +1)}-b\right) \end{array}$$

By considering the properties of *ϵ*, which is mentioned above, we can find that the right inequality is equal to the condition, with *R*_0_ close enough to 1.

When *c*_1_ < 0 and *c*_2_ > 0, *I*_*min*_ ≔ *argmin* *P*(*I*) is positive and when *R*_0_ = 1, there are two solutions for the quadratic Eq (10): *I*_1_ = 0 and *I*_2_ > 0. Therefore, when *R*_0_ = 1, *P*(*I*_*min*_)<0. Now by decreasing *R*_0_, when *R*_0_ is close enough to 1 and the conditions *c*_1_ < 0 and *P*(*I*_*min*_)<0 are held, there must be two positive solutions for the quadratic Eq (10). (The condition *P*(*I*_*min*_)<0 can be held because of continuity.) In this way, the second case of the theorem can be proved.

**Corollary 0.1**. *When R*_0_ < 1, *E*_0_ *is a unique fixed point if* $b>\frac{d}{(\delta +1)\beta }$ and there are two other fixed points X* *if* $b<\frac{d}{(\delta +1)\beta }$.

## Stability of fixed points

First, we must determine *Df* in order to analyze the stability of fixed points. *Df* is expressed as follows:

$$\begin{array}{c} Df\left(X\right)=(\begin{array}{cc} -d-\beta I & -\beta S\\ \beta I & \beta S-\delta -\frac{b^{2}}{{\left(I+b\right)}^{2}} \end{array}) \end{array}$$ (11)

Now *Df*(*E*_0_) can be expressed as follows:

$$\begin{array}{c} Df\left(E_{0}\right)=(\begin{array}{cc} -d & -\frac{\beta A}{d}\\ 0 & \frac{\beta A}{d}-\left(\delta +1\right) \end{array})=(\begin{array}{cc} -d & -\frac{\beta A}{d}\\ 0 & \left(\delta +1\right)\left(R_{0}-1\right) \end{array}) \end{array}$$ (12)

Therefore, the following theorem is resulted:

**Theorem 0.4**. *E*_0_ *is a stable node if R*_0_ < 1 *or is a saddle point if R*_0_ > 1.

*Proof*. As *Df*(*E*_0_) is an upper triangular matrix, its eigenvalues are −*d* and (*δ* + 1)(*R*_0_ − 1). Now if *R*_0_ < 1, then there are two negative eigenvalues and *E*_0_ will be a stable node; and if *R*_0_ > 1, then there is one negative and one positive eigenvalue and *E*_0_ will be a saddle point.

When other fixed points X* exist, *Df*(X*) can be expressed as follows:

$$\begin{array}{c} Df\left(X^{*}\right)=(\begin{array}{cc} -d-\beta I & -(\delta +\frac{b}{I+b})\\ \beta I & \frac{b}{I+b}-\frac{b^{2}}{{\left(I+b\right)}^{2}} \end{array})=(\begin{array}{cc} -d-\beta I & -(\delta +\frac{b}{I+b})\\ \beta I & \frac{bI}{{\left(I+b\right)}^{2}} \end{array}) \end{array}$$ (13)

Now the trace of *Df*(X*), which is the sum of the eigenvalues of *Df*(X*), is expressed as follows:

$$\begin{array}{c} \lambda_{1}+\lambda_{2}\left(I\right)=tr\left(Df\left(X^{*}\right)\right)=\frac{Ib}{{\left(I+b\right)}^{2}}-d-\beta I \end{array}$$

The determinant of *Df*(X*), which is the product of the eigenvalues of *Df*(X*), can be expressed by the following equations:

$$\begin{array}{cc} \lambda_{1}\lambda_{2}\left(I\right)=det\left(Df\left(X^{*}\right)\right) & =\beta I\left(\delta +\frac{b}{I+b}\right)-\frac{dbI}{{\left(I+b\right)}^{2}}-\frac{b\beta I^{2}}{{\left(I+b\right)}^{2}}\\ & =\frac{I}{{\left(I+b\right)}^{2}}\left(\beta \left(\delta {\left(I+b\right)}^{2}+b\left(I+b\right)\right)-db-\beta bI\right)\\ & =\frac{\beta I}{{\left(I+b\right)}^{2}}\left(\delta I^{2}+2b\delta I+b^{2}\left(\left(\delta +1\right)-\frac{d}{b\beta }\right)\right) \end{array}$$

There are the following theorems about the stability of fixed points X*:

**Theorem 0.5**. *When R*_0_ > 1, the unique fixed point X* *is stable if* $\frac{1}{4}<d$ *and* $\frac{d}{(\delta +1)\beta }<b$.

*Proof*. First, the necessary condition, which must be satisfied in both of the real and complex eigenvalues cases, is λ_1_ + λ_2_ < 0, i.e:

$$\begin{array}{c} \frac{Ib}{{\left(I+b\right)}^{2}}-d-\beta I<0 \end{array}$$

It is now easy to realize that the function $\frac{Ib}{{\left(I+b\right)}^{2}}$ is less than or equal to $\frac{1}{4}$ for non-negative *I*, and as we saw in the theorem, there is a fixed point with *I* ∈ (0, +∞) when *R*_0_ > 1. Therefore, the above inequality must be satisfied for ∀*I* ∈ (0, +∞), so:

$$\begin{array}{cc} \frac{1}{4}<d & \Rightarrow -d<-\frac{1}{4}\\ \Rightarrow \frac{Ib}{{\left(I+b\right)}^{2}}-d & <\frac{Ib}{{\left(I+b\right)}^{2}}-\frac{1}{4}<0\\ \Rightarrow \frac{Ib}{{\left(I+b\right)}^{2}} & -d-\beta I<0 \end{array}$$

However, when the eigenvalues of *Df*(X*) are real, another condition that must be satisfied is λ_1_λ_2_ > 0, i.e:

$$\begin{array}{c} \frac{\beta I}{{\left(I+b\right)}^{2}}\left(\delta I^{2}+2b\delta I+b^{2}\left(\left(\delta +1\right)-\frac{d}{b\beta }\right)\right)>0\;\forall I\in \left(0,+\infty \right) \end{array}$$

One can observe that the above inequality is satisfied in this interval if and only if:

$$\begin{array}{cc} b^{2}\left(\left(\delta +1\right)-\frac{d}{b\beta }\right) & >0\Rightarrow \\ \left(\delta +1\right)-\frac{d}{b\beta } & >0\Rightarrow \\ \frac{d}{(\delta +1)\beta } & <b \end{array}$$

And this completes the proof of this theorem.

## Bifurcations

In this section, it is shown that the system (6) undergoes three kinds of bifurcation, forward and backward bifurcations and Hopf bifurcation.

### Forward & backward bifurcation

**Theorem 0.6 (forward bifurcation)**. *When R*_0_ = 1, *the system* (6) *undergoes a forward bifurcation if* $\frac{1}{4}<d$ *and* $\frac{d}{(\delta +1)\beta }<b$.

*Proof*. According to the theorems of the previous sections, when $\frac{d}{(\delta +1)\beta }<b$, *E*_0_ is a unique stable fixed point for *R*_0_ < 1 and when $\frac{1}{4}<d$ and $\frac{d}{(\delta +1)\beta }<b$, there is a unique stable fixed point X* for *R*_0_ > 1 and *E*_0_ is an unstable fixed point. Therefore, when $\frac{d}{(\delta +1)\beta }<b$ and $\frac{1}{4}<d$, both of the conditions are satisfied and there is a forward bifurcation in the system (6).

**Fig 2:**
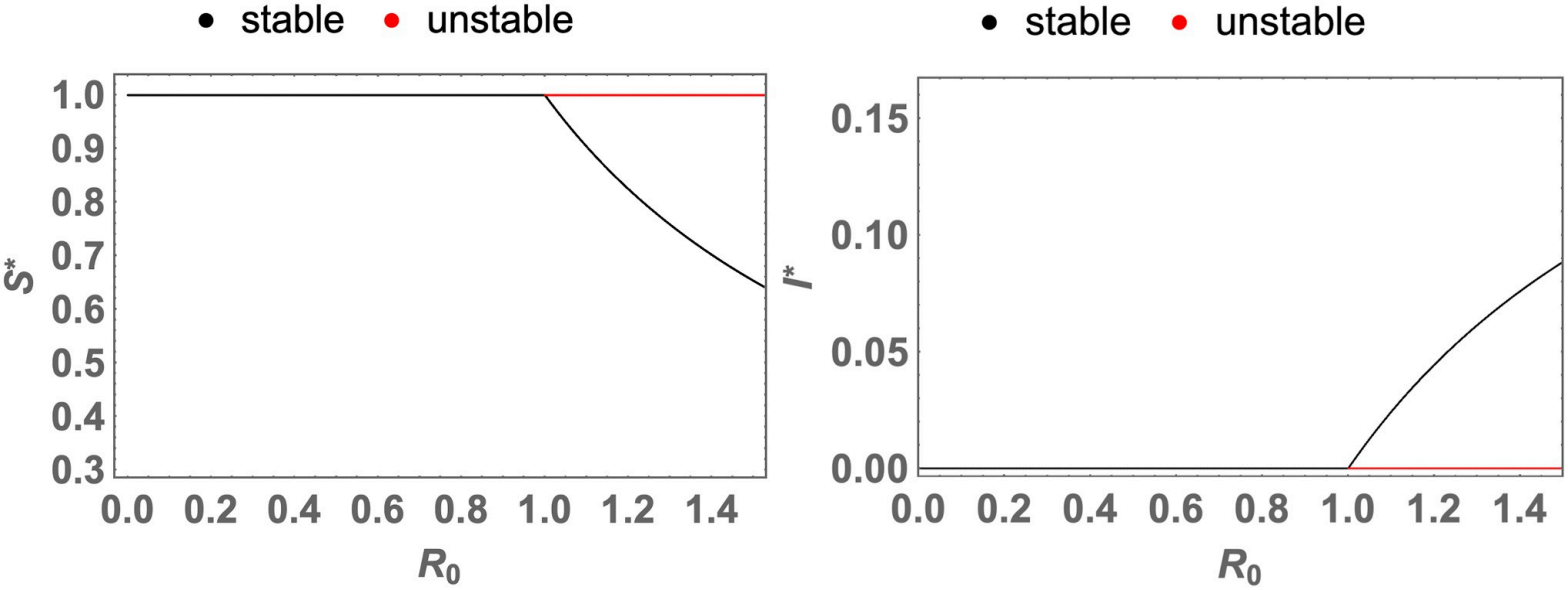
The simulation of the theorem 0.6 (forward bifurcation). The plots of S* and I*, which are the coordinates of the fixed points, as functions of *R*_0_ where the black and red curves respectively indicate the stable and unstable fixed points, and when the parameters are *b* = 1, *A* = 1, *d* = 1, *α* = 1, *n* = 1, and *n*′ = 1 and when *β* changes. The quantities and the parameters are defined in the Tables 1 and 2; *R*_0_ is defined in the Eq (9).

As an example, this theorem is demonstrated in the plots of Fig 2.

**Theorem 0.7 (backward bifurcation)**. *The system* (6) *undergoes a backward bifurcation for some R*_0_ < 1 *if* $\frac{1}{4}<d$ *and* $b<\frac{d}{(\delta +1)\beta }$.

**Fig 3:**
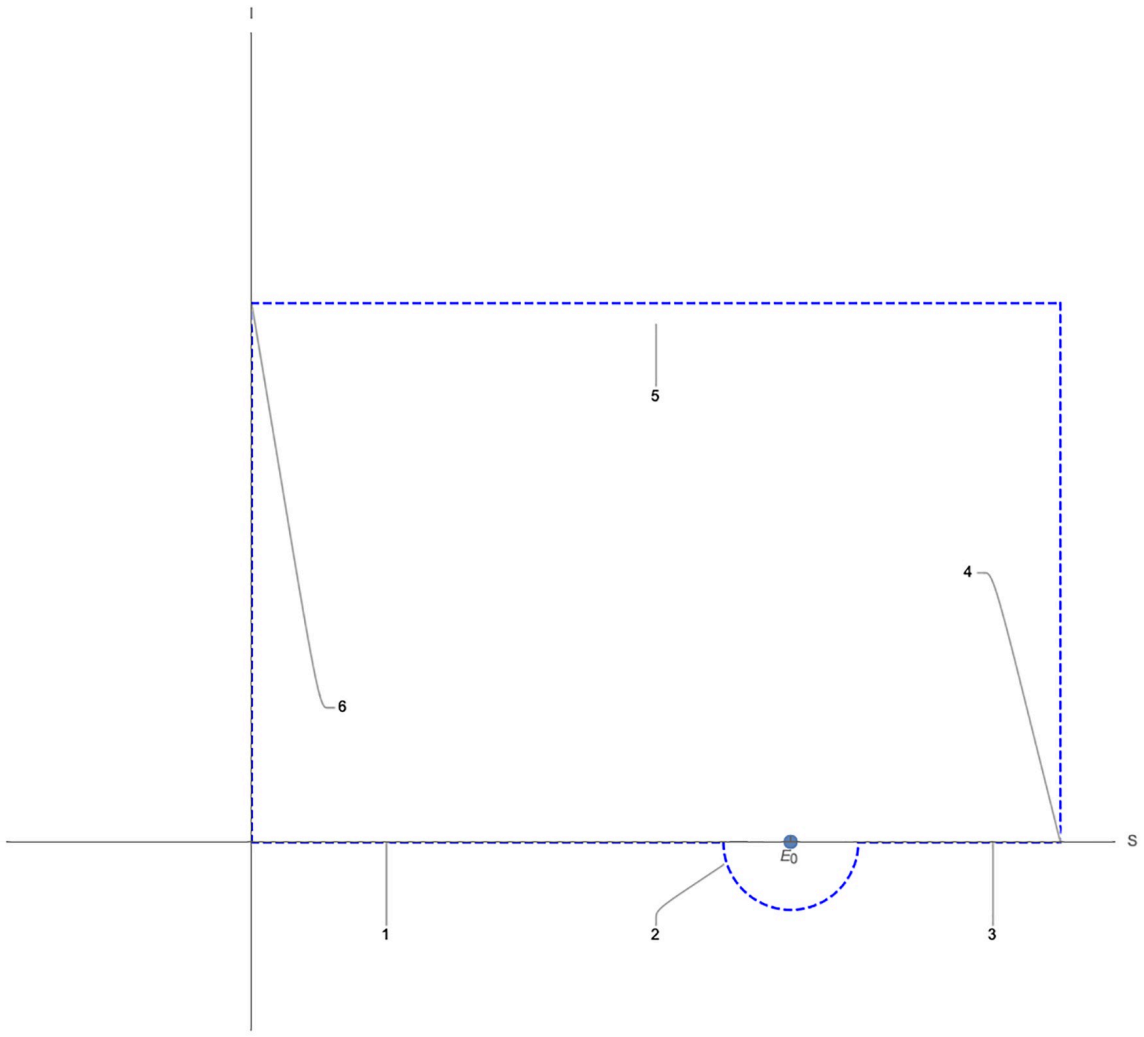
The closed curve *C*.

*Proof*. According to the theorems in the previous sections, when *R*_0_ < 1, *E*_0_ is always a stable fixed point and there are two other fixed points X* if $b<\frac{d}{(\delta +1)\beta }$ and *R*_0_ close enough to 1. Now we claim that one of these fixed points X* is a stable node and the other one is a saddle point. We prove this by considering the *Index Theory*. First, we choose one closed curve *C* like in Fig 3:

This curve *C* is chosen large enough, so when the fixed points X* exist, this curve *C* encloses them. (The semicircle in the fourth quadrant is too small, so it encloses just *E*_0_ for *R*_0_ < 1).

Now when *R*_0_ < 1 and small, there is just *E*_0_ in the curve *C*, so the index of the closed curve *C* is as follows:

$$\begin{array}{c} I_{C}=I_{0} \end{array}$$

where *I*_0_ is the index of *E*_0_. *E*_0_ is a stable node, so *I*_0_ = 1 and:

$$\begin{array}{c} I_{C}=1 \end{array}$$

Now we assume that *R*_0_ is close enough to 1 and that two other fixed points X* exist in the *C*. Therefore, *I*_*C*_ is expressed as follows:

$$\begin{array}{c} I_{C}=I_{0}+I_{1}+I_{2}=1+I_{1}+I_{2} \end{array}$$

where *I*_1_ and *I*_2_ are the indices of the other fixed points X*. Thus *I*_*C*_ can be considered as a function of *R*_0_.

Now we claim that the *I*_*C*_(*R*_0_) is a continuous function. *I*_*C*_(*R*_0_) can be rewritten as an integral in the complex plane as follows:

$$\begin{array}{c} I_{C}\left(R_{0}\right)=\frac{1}{2\pi i}\oint_{\tilde{C}}\;\frac{1}{\xi }\;\text{d}\xi \end{array}$$

where $\tilde{C}$ is *f*(*C*)(*C* is considered as a closed curve in C and f as a function C→C). As a result, we can obtain:

$$\begin{array}{c} I_{C}\left(R_{0}\right)=\frac{1}{2\pi i}\sum_{i=1}^{6}\int_{0}^{1}\;\frac{1}{f(g_{i}\left(t\right))}f^{\prime }\left(g_{i}\left(t\right)\right)g_{i}^{\prime }\left(t\right)\;dt \end{array}$$

where *g*_*i*_(*t*) indicates the parametrization of the lines and the semicircle of the closed curve *C* in the complex plane, as it is shown in Fig 3.

Now *f* and *f*′ are continuous functions of *R*_0_, so the above integrands are continuous functions of *R*_0_, and *I*_*C*_(*R*_0_) is a continuous function of *R*_0_.

We know that *I*_1,2_ = ±1 and *I*_*C*_(*R*_0_) is continuous, so:

$$\begin{array}{cc} & \left\{ \begin{array}{cc} I_{C}\left(R_{0}\right)=1\; & R_{0}\in \left(0,R_{0}^{\left(1\right)}\right)\\ I_{C}\left(R_{0}\right)=1+I_{1}+I_{2}\; & R_{0}\in \left(R_{0}^{\left(1\right)},1\right) \end{array}\right.\\ \Rightarrow & I_{1}+I_{2}=0\\ \Rightarrow & \left\{ \begin{array}{c} I_{1}=1\;\&\;I_{2}=-1\\ or\\ I_{2}=1\;\&\;I_{1}=-1 \end{array}\right. \end{array}$$

As a result, one of the fixed points X* must be a saddle point (*I* = −1) and the other one must be a stable or an unstable node (*I* = 1), but $d>\frac{1}{4}$ and λ_1_ + λ_2_ < 0, so it must be a stable node.

Now when *R*_0_ > 1, *E*_0_ is an unstable fixed point (saddle point) and there is always an other fixed point $X_{1}^{*}$ in the first quadrant and a fixed point $X_{2}^{*}$ near *E*_0_ in the semicircle($X_{2}^{*}$ is not a permissible fixed point because it exists in the fourth quadrant). Therefore, *I*_0_ = −1 and *I*_*C*_(*R*_0_) is continuous:

$$\begin{array}{cc} & \left\{ \begin{array}{cc} I_{C}\left(R_{0}\right)=1\; & R_{0}\in \left(0,1\right)\\ I_{C}\left(R_{0}\right)=-1+I_{1}+I_{2}\; & R_{0}\in \left(1,+\infty \right) \end{array}\right.\\ \Rightarrow & I_{1}+I_{2}=2\\ \Rightarrow & I_{1,2}=1 \end{array}$$

As a result, $X_{1,2}^{*}$ are nodes. However, it is similar to the case *R*_0_ < 1 and the permissible node must be stable, so $X_{1}^{*}$ is a stable fixed point.

Therefore, there is just a stable fixed point (*E*_0_) for $R_{0}<R_{0}^{\left(1\right)}$ and two stable fixed points($E_{0},X_{1}^{*}$) and an unstable fixed point ($X_{2}^{*}$) for $R_{0}^{\left(1\right)}<R_{0}<1$ and a stable fixed point $X_{1}^{*}$ and an unstable fixed point *E*_0_ for *R*_0_ > 1. There must hence be a backward bifurcation when $R_{0}=R_{0}^{\left(1\right)}<1.$

**Fig 4:**
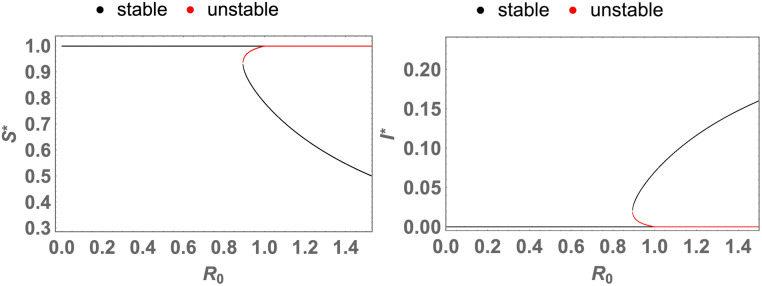
The simulation of the theorem 0.7 (backward bifurcation). The plots of S* and I*, which are the coordinates of the fixed points, as functions of *R*_0_ where the black and red curves respectively indicate the stable and unstable fixed points, and when the parameters are *b* = .01, *A* = 1, *d* = 1, *α* = 1, *n* = 1, and *n*′ = 1, and when *β* changes. The quantities and the parameters are defined in the Tables 1 and 2; *R*_0_ is defined in the Eq (9).

As an example, this theorem is illustrated in the plots of Fig 4.

**remark 0.3**. The saddle node bifurcation in backward bifurcation occurs when the quadratic Eq (10) has just one root, a.e:

$$\begin{array}{c} \Delta =0\Leftrightarrow c_{1}^{2}-4c_{2}c_{0}=0 \end{array}$$ (14)

Now when *β* is changing and other parameters are constant, *β* can be obtained form the above equation. For example, *β* = 0.892857 in the simulation of previous theorem, Fig 4.

### Hopf bifurcation

**remark 0.4**. In the following theorem, *R*_0_ is considered as a function of *β*, and the other parameters are constant. In other words, as *R*_0_ ∝ *β*, there is no difference between changing of *R*_0_ and *β*. It is advisable to define *β*_0_ as follows:

$$\begin{array}{cc} & \beta_{0}≔\frac{(\delta +1)d}{A}\\ \Rightarrow & R_{0}=1\Leftrightarrow \beta =\beta_{0} \end{array}$$

**Theorem 0.8 (Hopf bifurcation)**. *Suppose* $\frac{b(2\delta +1)}{2}<A$ *and* $\frac{d}{(\delta +1)\beta }<b$. *Define* Δ *and β*_*max*_ *as follows*:

$$\begin{array}{c} \Delta ≔A-\frac{b(2\delta +1)}{2}\;\beta_{max}≔\frac{(\delta +1)d}{\Delta } \end{array}$$

*By considering the above assumptions, the system* (6) *undergoes a Hopf bifurcation for some β* ∈ (*β*_0_, *β*_*max*_) *or equally for some* $R_{0}\in \left(1,\frac{A}{\Delta }\right)$ *if* $d\left(1+\frac{(\delta +1)b}{\Delta }\right)<\frac{1}{4}$.

*Proof*. First, we consider λ_1_ + λ_2_(*I*). We have shown:

$$\begin{array}{c} \lambda_{1}+\lambda_{2}\left(I\right)=\frac{Ib}{{\left(I+b\right)}^{2}}-d-\beta I \end{array}$$

At First, we fix *β* ∈ [*β*_0_, *β*_*max*_]. Now when *I* = 0, λ_1_ + λ_2_ = −*d* < 0 and when *I* = *b*:

$$\begin{array}{c} \lambda_{1}+\lambda_{2}\left(b\right)=\frac{1}{4}-d-\beta b \end{array}$$

we will have:

$$\begin{array}{cc} \beta & \leq \beta_{max}\\ \Rightarrow \frac{1}{4}-d-\beta b & \geq \frac{1}{4}-d-b\beta_{max}\\ \Rightarrow \frac{1}{4}-d-\beta b & \geq \frac{1}{4}-d-\frac{(\delta +1)db}{\Delta }\\ \Rightarrow \frac{1}{4}-d-\beta b & \geq \frac{1}{4}-d\left(1+\frac{(\delta +1)b}{\Delta }\right)>0\\ \Rightarrow \lambda_{1}+\lambda_{2}\left(b\right) & =\frac{1}{4}-d-\beta b>0 \end{array}$$

By considering the above assumptions, for each *β* ∈ [*β*_0_, *β*_*max*_], we have λ_1_ + λ_2_(0)<0 and λ_1_ + λ_2_(*b*)>0. Therefore, and according to the *intermediate value theorem*, for each *β* ∈ [*β*_0_, *β*_*max*_], there is some *I*_1_ ∈ (0, *b*) such that λ_1_ + λ_2_(*I*_1_) = 0. However, if we consider *I*_1_ as the intersection point of the function $\frac{Ib}{{\left(I+b\right)}^{2}}$ and the line *βI* + *d*, and by considering the behavior of the function and line, there is just one *I*_1_ for each *β* ∈ [*β*_0_, *β*_*max*_] and *I*_1_(*β*) is continuous and 0 < *I*_1_(*β*)<*b* and λ_1_ + λ_2_(*I*_1_(*β*)) = 0 when *β* ∈ [*β*_0_, *β*_*max*_].

Now we consider the quadratic Eq (10). When $\frac{d}{(\delta +1)\beta }<b$ and *R*_0_ = 1 or equally *β* = *β*_0_, there is just one permissible solution: *I* = 0; and when *R*_0_ > 1, there is always just one permissible root that is a continuous function of *R*_0_ or other parameters like *β* (*I*(*β*)). Now we consider the root of the quadratic Eq (10) for *β* = *β*_*max*_. In this case, the quadratic Eq (10) is expressed as follows:

$$\begin{array}{c} P_{M}\left(I\right)=\delta I^{2}+\left(b\left(\delta +1\right)-A\right)I-bA+\frac{\delta d}{\beta_{max}}I+\frac{bd}{\beta_{max}}\left(\delta +1\right) \end{array}$$

We then consider *P*_*M*_(*b*) and the condition $\frac{b(2\delta +1)}{2}<A$:

$$\begin{array}{cc} P_{M}\left(b\right) & =\delta b^{2}+\left(b\left(\delta +1\right)-A\right)b-bA+\frac{\delta db}{\beta_{max}}+\frac{bd}{\beta_{max}}\left(\delta +1\right)\\ & =b\left(\delta b+b\left(\delta +1\right)-2A\right)+b\left(\frac{\delta d}{\beta_{max}}+\frac{d}{\beta_{max}}\left(\delta +1\right)\right)\\ & =-2b\left(A-\frac{b(2\delta +1)}{2}\right)+\frac{b(2\delta +1)d}{\beta_{max}}\\ & =b(-2\Delta +\frac{(2\delta +1)\Delta }{\delta +1})\\ & =b\Delta (-2+\frac{2\delta +1}{\delta +1})\\ & =b\Delta (\frac{-1}{\delta +1})<0 \end{array}$$

Obviously, *P*_*M*_(*I*) has a root *I* in the interval (*b*, +∞). In other words, by assuming the conditions $\frac{d}{(\delta +1)\beta }<b$ and $\frac{b(2\delta +1)}{2}<A$, the quadratic Eq (10) has a root *I* = 0 when *β* = *β*_0_ and a root *I* > *b* when *β* = *β*_*max*_ and the I*(*β*) is a continuous function of *β* ∈ [*β*_0_, *β*_*max*_]. Next we use the *intermediate value theorem* for the function *f*(*β*) = *I*_1_ − I*(*β*) and *β* ∈ [*β*_0_, *β*_*max*_]. This function is obviously continuous and

$$\begin{array}{cc} & f\left(\beta_{0}\right)=I_{1}\left(\beta_{0}\right)-I^{*}\left(\beta_{0}\right)=I_{1}\left(\beta_{0}\right)>0\\ & f\left(\beta_{max}\right)=I_{1}\left(\beta_{max}\right)-I^{*}\left(\beta_{max}\right)<0 \end{array}$$

As a result, there is a *β*_1_ ∈ (*β*_0_, *β*_*max*_) such that *f*(*β*_1_) = 0 or equally *I*_1_(*β*_1_) = I*(*β*_1_) and

$$\begin{array}{c} \lambda_{1}+\lambda_{2}\left(I^{*}\left(\beta_{1}\right)\right)=\lambda_{1}+\lambda_{2}\left(I_{1}\left(\beta_{1}\right)\right)=0 \end{array}$$

By considering the differentiability of *I*(*β*) and λ_1_ + λ_2_(*I*) and the *intermediate value theorem*, it is possible to find a *β*_1_ with the following properties:

$$\begin{array}{c} \left(\exists \epsilon >0\right)\;I\left(\beta_{1}\right)=I_{1}\left(\beta_{1}\right)\;,\{\begin{array}{c} \lambda_{1}+\lambda_{2}\left(I\left(\beta \right)\right)<0\;\beta \in \left(\beta_{1}-\epsilon ,\beta_{1}\right)\\ \lambda_{1}+\lambda_{2}\left(I\left(\beta \right)\right)=0\;\beta =\beta_{1}\\ \lambda_{1}+\lambda_{2}\left(I\left(\beta \right)\right)>0\;\beta \in \left(\beta_{1},\beta_{1}+\epsilon \right) \end{array} \end{array}$$

We now consider λ_1_λ_2_(*I*). We have proved that λ_1_λ_2_(*I*) is positive when *I* ∈ (0, +∞) and $\frac{d}{(\delta +1)\beta }<b$, so:

$$\begin{array}{c} \left\{ \begin{array}{c} \lambda_{1}+\lambda_{2}\left(I\left(\beta_{1}\right)\right)=0\\ \lambda_{1}\lambda_{2}\left(I\left(\beta_{1}\right)\right)>0 \end{array}\Rightarrow -\lambda_{1}^{2}>0\Rightarrow \{\begin{array}{c} \lambda_{1}\left(I\left(\beta_{1}\right)\right)=i\omega_{0}\\ \lambda_{2}\left(I\left(\beta_{1}\right)\right)=-i\omega_{0} \end{array},\omega_{0}>0\right. \end{array}$$

Obviously, λ_1,2_ are continuous functions of *I* and *β*, and *I* is a continuous function of *β*, so λ_1,2_(*β*) is continuous with values in the complex plane C. Consequently, we can select a *ϵ*, as defined in the above arguments, with the following properties:

$$\begin{array}{c} Im\left(\lambda_{1}\left(I\left(\beta \right)\right)\right)>0\;,\beta \in \left(\beta_{1}-\epsilon ,\beta_{1}+\epsilon \right) \end{array}$$

As $\lambda_{2}={\bar{\lambda }}_{1}$, the above results can be summarized and rewritten as follows:

$$\begin{array}{cc} {}[\left(\exists \epsilon >0\right)\forall \beta \in (\beta_{1}-\epsilon & ,\beta_{1}+\epsilon )]\;\lambda_{1,2}\left(\beta \right)=r\pm i\omega \;,\omega >0\\ , & \left\{ \begin{array}{c} r<0\;\beta \in (\beta_{1}-\epsilon ,\beta_{1})\\ r=0\;\beta =\beta_{1}\\ r>0\;\beta \in (\beta_{1},\beta_{1}+\epsilon ) \end{array}\right. \end{array}$$

As a result, there must be a Hopf bifurcation when *β* = *β*_1_ ∈ (*β*_0_, *β*_*max*_) or equally $R_{0}=\frac{\beta_{1}A}{(\delta +1)\beta }\in \left(1,\frac{A}{\Delta }\right).$

**Fig 5:**
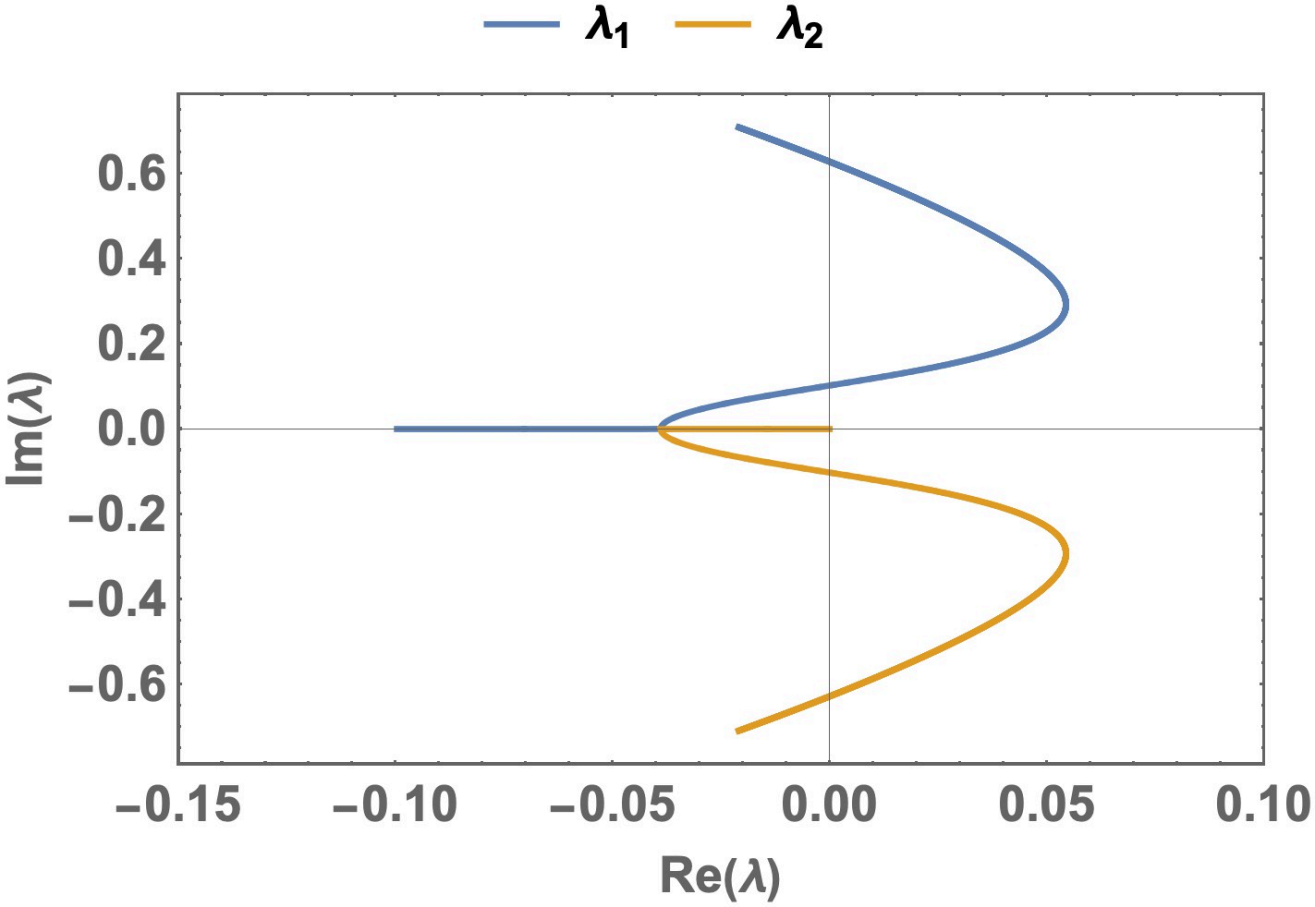
The simulation of the theorem 0.8 (Hopf bifurcation). The curves of the eigenvalues in the complex plane where the blue and yellow curves indicate different eigenvalues and when the parameters are *A* = 10, *d* = .1, *α* = 1.9, *n* = 1, and *b* = 1, and when *β* changes in the interval (.04, .09) or equally *R*_0_ ∈ (1, 2.25). The quantities and the parameters are defined in the Tables 1 and 2; *R*_0_ is defined in the Eq (9).

As an example, this theorem can be observe in the plot of Fig 5. It is straightforward to examine that these parameters satisfy the conditions of this theorem, and *β*_0_ = .04 and *β*_*max*_ ≈.06154. Obviously, there are two Hopf bifurcations in this case. These bifurcations occur as *β*_1_ ≈ 0.040968 and *β*_2_ ≈ 0.078668 or equally as $R_{0}^{\left(1\right)}\approx 1.0242$ and $R_{0}^{\left(2\right)}\approx 1.9667$. The case *β*_1_ was predicted in our theorem.

## Numerical analysis

**Fig 6:**
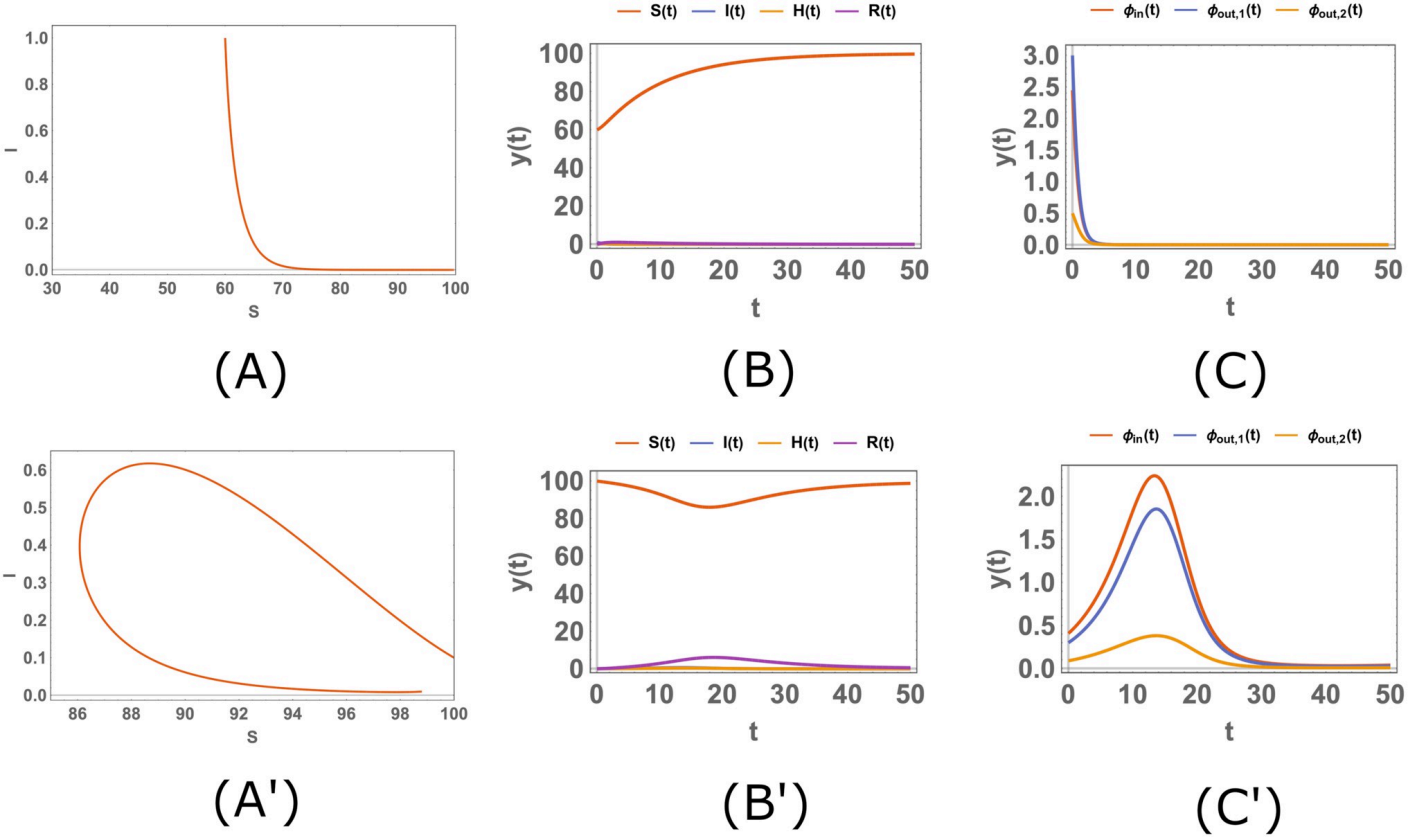
The simulation of the system (5). The stream plot *S* − *I* (*A*, *A*′) and the plots of *S*, *I*, *H*, *R* (*B*, *B*′) and the fluxes (*C*, *C*′) as functions of *t* when the parameters are *b* = 1, *A* = 10, *d* = .1, *α* = 1.9, *n* = 1, *n*′ = 1 and when *β* = .0408; the initial conditions are *X*_0_ = (60, 1, 0, 0) For panels (*A*, *B*, *C*) and *X*_0_ = (100, .1, 0, 0) (*A*′, *B*′, *C*′). The quantities and the parameters are defined in the Tables 1 and 2; the fluxes are defined in the Eq (15).

**Fig 7:**
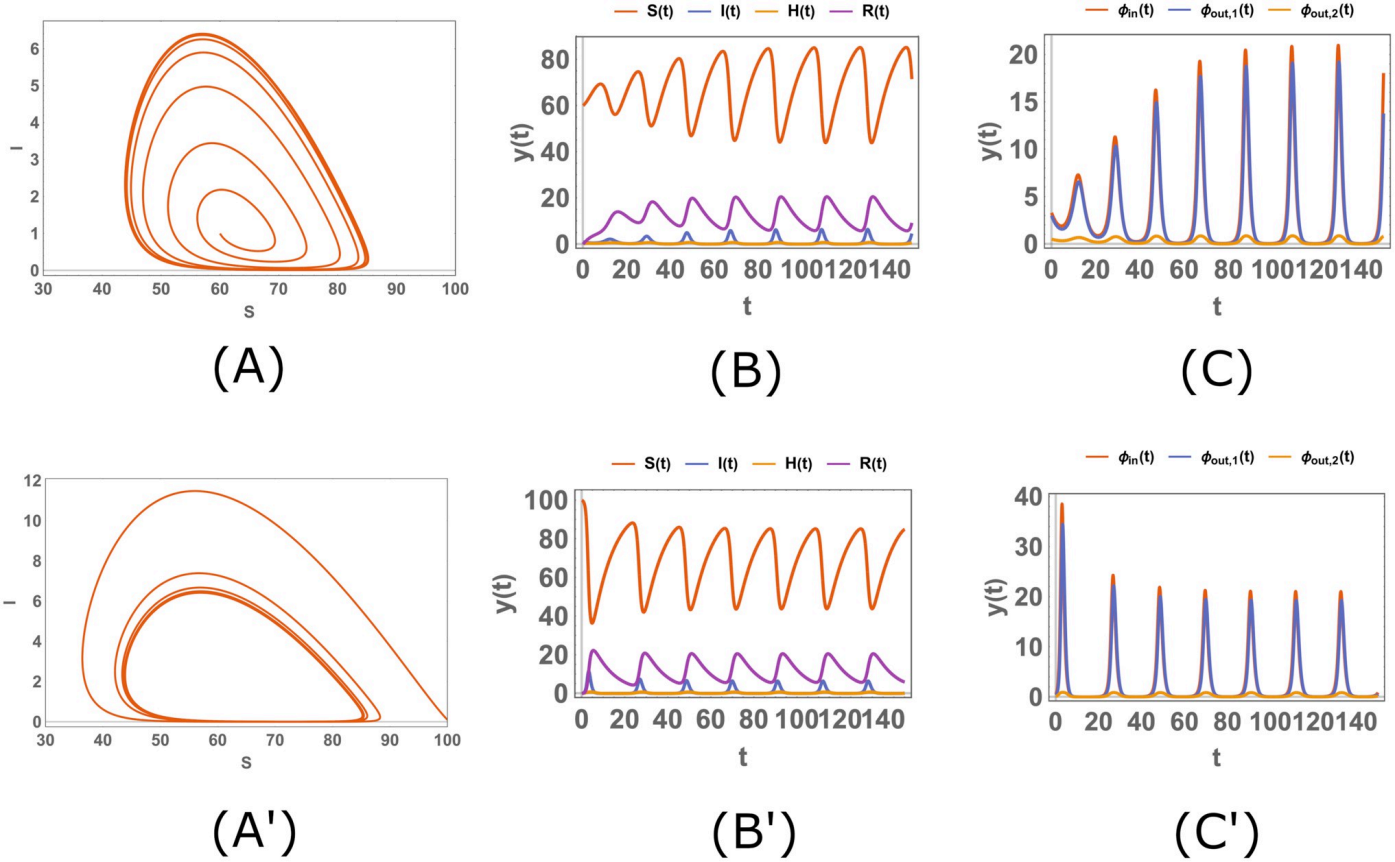
The simulation of the system (5). The stream plot *S* − *I* (*A*, *A*′) and the plots of *S*, *I*, *H*, *R* (*B*, *B*′) and the fluxes (*C*, *C*′) as functions of *t* when the parameters are *b* = 1, *A* = 10, *d* = .1, *α* = 1.9, *n* = 1, *n*′ = 1 and when *β* = .055; the initial conditions are *X*_0_ = (60, 1, 0, 0) for panels (*A*, *B*, *C*) and *X*_0_ = (100, .1, 0, 0) for panels (*A*′, *B*′, *C*′). The quantities and the parameters are defined in the Tables 1 and 2; the fluxes are defined in the Eq (15).

In this section, we concentrate on a range of the parameters where there is a Hopf bifurcation, and we subsequently analyze the time evolution of the model. For instance, we can consider the case in Fig 5, where *β* changes in the interval (.04, .09) with obviously one limit cycle in this situation for *β* ∈ (0.040968, 0.078668). Now we choose two different values for *β*, before (Fig 6) and after (Fig 7) *β*_1_ ≈ 0.040968, with two different initial conditions for each *β*, and we sketch the stream plot *S* − *I* and *S*, *I*, *H*, *R* and the fluxes as functions of t, where the fluxes are defined as follows:

$$\begin{array}{c} \left\{ \begin{array}{c} \phi_{in}≔\beta IS\\ \phi_{out,1}≔\left(d+\alpha +n\right)I\\ \phi_{out,2}≔\frac{bI}{I+b} \end{array}\right. \end{array}$$ (15)

In Fig 6, *β* is less than 0.040968, so there is no limit cycle in this condition and the curves in the stream plots (panels (*A*, *A*′)) approach a fixed point; therefore, the plots of *S*, *I*, *H*, *R* and the fluxes have a limit when *t* → ∞. However, in Fig 7, *β* > 0.040968 and there is a limit cycle and the curves in the stream plots (panels (*A*, *A*′)) approach this limit cycle. As a result, the plots of *S*, *I*, *H*, *R* and the fluxes behave periodically when *t* is large enough.

## Discussion & conclusion

In our study, we modified the standard SIR model and divided the infected individuals who survived in the block *R* and in a newly proposed block *H* in order to model the number of individuals hospitalized in an ICU with their hospitalization rates defined respectively as *nI* and $\frac{bI}{I+b}$. Our model can be considered as a specific version of the model developed by Zhang et al. [14], but we concentrated on the existence and types of bifurcation, and we could observe the forward, backward and Hopf bifurcations.

The most crucial finding of our study is that we demonstrated the existence of Hopf bifurcation and the limit cycle. The existence of the limit cycle, in particular, illustrates that a disease can survive in a community and that this is a potentially critical situation for governments in controlling the spread of a disease.

As an application of our model, we imagine the following case based on the existence of Hopf bifurcation. Suppose that we are in a range of the parameters where a supercritical Hopf bifurcation can occur when *β* = *β*_0_, resembling the case considered in the numerical analysis section. Once a given disease spreads, governments usually utilize interventions in order to reduce contact among people, for instance, by implementing quarantine measures. As a result, *β* is small and we can suppose that *β* < *β*_0_ where there is no limit cycle. However, after a while, it is possible that *β* increases and *β* > *β*_0_. This event is highly probable to occur, for example, when people ignore interventions of governments and quarantine requirements. There exists, in this situation, a limit cycle and the disease can survive.

A limitation of our study is that we did not analyze the period of limit cycle and the time evolution of the fluxes, which were defined in the Eq (15), which can be researched by numerical analysis more precisely and more convenient instead of real analysis. It is worth mentioning that the fluxes are crucial since they are practically observable in hospitals and can be deployed as a sort of warning signal. Moreover, these fluxes can help estimate the other parameters of the disease spread dynamics. Finally, the proved theorems and the introduced fluxes provide suitable materials for future numerical researches such as global stability, sensitivity, optimal control, which can involve real situations and data of real infection.

## Supporting information

S1 File (ZIP)
